# Modulation of Cadmium Tolerance in Rice: Insight into Vanillic Acid-Induced Upregulation of Antioxidant Defense and Glyoxalase Systems

**DOI:** 10.3390/plants9020188

**Published:** 2020-02-04

**Authors:** M.H.M. Borhannuddin Bhuyan, Khursheda Parvin, Sayed Mohammad Mohsin, Jubayer Al Mahmud, Mirza Hasanuzzaman, Masayuki Fujita

**Affiliations:** 1 Laboratory of Plant Stress Response, Department of Applied Biological Sciences, Faculty of Agriculture, Kagawa University, Miki-Cho, Kita-gun, Kagawa 761-0795, Japanhirasau@gmail.com (K.P.); mohsinsau.ac@gmail.com (S.M.M.); 2 Citrus Research Station, Bangladesh Agricultural Research Institute, Jaintapur, Sylhet 3156, Bangladesh; 3 Department of Horticulture, Sher-e-Bangla Agricultural University, Dhaka 1207, Bangladesh; 4 Department of Plant Pathology, Sher-e-Bangla Agricultural University, Dhaka 1207, Bangladesh; 5 Department of Agroforestry and Environmental Science, Sher-e-Bangla Agricultural University, Dhaka 1207, Bangladesh; jamahmud_bd@yahoo.com; 6 Department of Agronomy, Sher-e-Bangla Agricultural University, Dhaka 1207, Bangladesh

**Keywords:** abiotic stress, antioxidant defense, metal toxicity, methylglyoxal, oxidative stress, organic acid

## Abstract

Cadmium (Cd) is a toxic heavy metal that enters the human food chain from the soil via plants. Increased Cd uptake and translocation in plants alters metabolism andreduces crop production. Maintaining crop yield therefore requires both soil remediation andenhanced plant tolerance to Cd. In this study, we investigated the effects of vanillic acid (VA) on Cd accumulation and Cd stress tolerance in rice (*Oryza sativa* L. cv. BRRI dhan54). Thirteen-day-old rice seedlings treated with CdCl_2_ (1.0 and 2.0 mM) for 72 h showed reduced growth, biomass accumulation, and water and photosynthetic pigment contents, as well as increased signs of oxidative stress (elevated levels of malondialdehyde, hydrogen peroxide, methylglyoxal, and lipoxygenase) and downregulated antioxidant and glyoxalase systems. Cadmium-induced changes in leaf relative turgidity, photosynthetic pigment content, ascorbate pool size, and glutathione content were suppressed by VA under both mild and severe Cd toxicity stress. The supplementation of VA under Cd stress conditions also increased antioxidant and glyoxylase enzyme activity. Vanillic acid also increased phytochelatin content and the biological accumulation factor, biological accumulation co-efficient, and Cd translocation factor. Vanillic acid, therefore appears to enhance Cd stress tolerance by increasing metal chelation and sequestration, by upregulating antioxidant defense and glyoxalase systems, and by facilitating nutrient homeostasis.

## 1. Introduction

The global human population is increasing rapidly, and feeding growing numbers of people has become a challenging task for farmers. Likewise, plant biologists face related challenges in their search for plant varieties that can produce sufficient food on farmlands that are increasingly prone to abiotic stresses that cause huge crop production losses. Among these abiotic stresses, metal/metalloid toxicity is one of the most common and most deleterious.

Metal/metalloid toxicities are largely a consequence of environmental pollution due to rapid industrialization. One of the most harmful industrial metal pollutants is cadmium (Cd), as it is highly toxic to virtually all life forms, including humans, animals, and plants, and it readily enters the trophic chain primarily via plants [1]. Cadmium is rapidly taken up by plants and accumulates in various tissues due to its very high mobility and hydrophilic nature [2]. Plants that accumulate Cd show stunted growth due to cadmium-induced toxicity, which can also induce chlorosis, epinasty, and disruptions in pollen germination and pollen tube growth. Cadmium stress can also cause alterations in chloroplast ultrastructure, thereby inhibiting photosynthesis and CO_2_ fixation, as well as disturbing N and S metabolism [3].

Cadmium is a redox-inactive metal in nature, and thus it does not generate reactive oxygen species (ROS) directly by the Haber–Weiss reaction [4]. Instead, it alters the function of electron transport chains and disrupts antioxidant activities, thereby creating oxidative stress conditions in plants [5]. Cadmium also has a strong affinity for protein side chains and nitrogenous bases, and thus it can damage proteins and nucleic acids to further alter oxidative phosphorylation processes [5]. The generation of ROS in living organisms is an unavoidable consequence of aerobic metabolism. These Cd-induced oxidative stress reactions indirectly lead to overproduction of ROS (singlet oxygen, ^1^O_2_; superoxide, O_2_^•−^; hydrogen peroxide, H_2_O_2_; hydroxyl radical, OH) and initiate chain reactions that damage important plant biomolecules [6]. However, plants are sessile organisms and cannot move away from environmental sources of toxic pollutants such as Cd. Instead, they must rely on specially developed biological mechanisms that allow them to tolerate the presence of toxic metals. These mechanisms involve processes for avoidance, exclusion, excretion, binding, chelation, and compartmentalization of Cd [7].

Plants have limited strategies for avoidance of Cd toxicity, but their inherent capacity for dealing with oxidative stress is also not sufficient to cope with metal-induced oxidative damage. The plant’s antioxidant defense system consists of antioxidants that are enzymatic (superoxide dismutase [SOD], catalase [CAT], ascorbate peroxidase [APX], monodehydroascorbate reductase [MDHAR], dehydroascorbate reductase [DHAR], glutathione reductase [GR], glutathione-*S*-transferase [GST], and glutathione peroxidase [GPX]) and nonenzymatic (ascorbic acid [AsA], glutathione [GSH], tocopherol, and phenolic compounds). These enzymatic and nonenzymatic antioxidants function to counteract ROS production and serve as cellular redox buffers [6]. Metal/metalloid toxicity, and especially Cd stress, also induces the production of a highly cytotoxic compound, methylglyoxal (MG), which causes structural damage to cellular components, destroys DNA, and creates mutations [8,9]. The damage from MG is diminished in plants through the action of the glyoxylase system, which consists of the glyoxalase I (Gly I) and glyoxalase II (Gly II) enzymes that detoxify MG [9].

Both enzymatic and nonenzymatic antioxidants work simultaneously to combat the deleterious effects of oxidative stress caused by exposure to abiotic factors such as Cd. However, another notable response to several abiotic stresses is a marked accumulation of phenolic compounds [10,11,12,13,14]. Some phenolics have the capability to scavenge ROS directly as a way to reduce oxidative stress [11]. In recent decades, plant biologists have uncovered numerous different phytoprotectants in the form of secondary metabolites, hormones, nutrients, and allelopathic compounds, which all offer advantages to plants under stress conditions [15]. Metabolic manipulation of these compounds in modern agriculture therefore has great potential to improve crop plant tolerance to abiotic stresses such as Cd pollution.

One crop plant that is particularly hampered by Cd toxicity is rice—the most important cereal crop that is consumed by the half of the world community as a staple food [4]. Rice has also been reported to show dramatic stress-related increases in phenolics, vanillic acid (VA) in particular, which increases sevenfold in rice seedlings subjected to flooding [11]. The aim of the present study was therefore to examine the effects of application of exogenous VA on rice seedlings under Cd stress, with a particular focus on the antioxidant defense and glyoxalase systems, as well as nutrient homeostasis during the early seedling stage. To the best of our knowledge, this study is the first to investigate improvements in Cd stress tolerance in rice by VA-induced modulation of ion homeostasis, antioxidant defenses, and the glyoxalase system.

## 2. Results

### 2.1. Vanillic Acid Prevented the Suppression of Growth and Biomass Accumulation under Cd Toxicity

**Figure 1:**
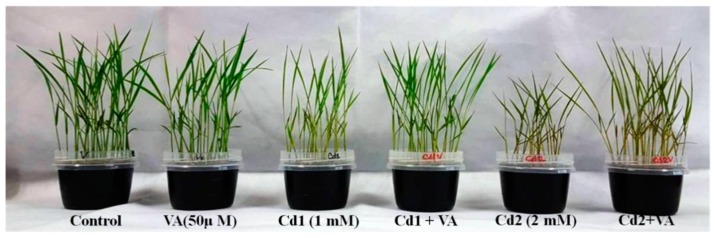
Visual images of the morphological differences in rice (*Oryza sativa* L. cv. BRRI dhan54) seedlings grown under different treatments: VA (50 µM vanillic acid), Cd1 (1.0 mM CdCl_2_), andCd2 (2.0 mM CdCl_2_).

Cadmium exposure resulted in a decrease in plant growth, visible as a reduction in plant height and root length as well as a limited relative elongation of the shoot (RSE) and root (RRE). Cadmium toxicity also reduced the overall seedling biomass (Figure 1).

Compared to the control condition, Cdstress (1.0 and 2.0 mM) reduced plant height by 14% and 23% and root length by 28 and 33%, respectively. The RSE was reduced by 53% and 79% and the RRE by 87% and 93%, respectively, by 1.0 and 2.0 mM CdCl_2_ (Table 1). A similar result was also observed for biomass accumulation. However, supplementation of the growth media with exogenous VA in the presence of Cd increased the shoot and root length, their relative elongation percentages, and biomass accumulation under both doses of Cd (Table 1; Figure 1).

### 2.2. Vanillic Acid–induced Accumulation, Translocation, and Detoxification of Cd

**Figure 2:**
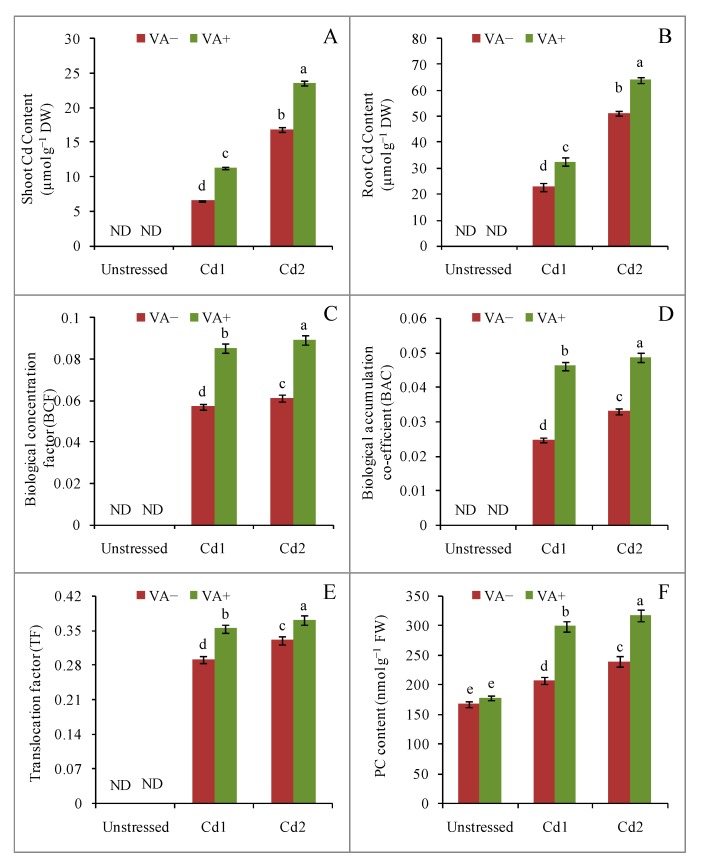
Effects of exogenous VA on Cd content of shoot (**A**), root (**B**), biological accumulation factor (BCF) (**C**), biological accumulation co-efficient (BAC) (**D**), translocation factor (TF) (**E**), and phytochelatin (PC) (**F**) contents of rice (*Oryza sativa* L. cv. BRRI dhan54) seedlings under Cd toxicity stress. Mean (±SD) was computed from three replications of each treatment. Bars with dissimilar letters are significantly different at *p* ≤ 0.05 from Fisher’s least significant difference (LSD) test.

Cadmium exposure induced a dose-dependent increase in the Cd content of the rice seedlings. Significant differences were noted in the Cd content of the shoots and roots, and Cd content was much higher in the root than in the shoot. Addition of VA to the growth medium further increased the Cd content in the shoot (Figure 2A) and the root (Figure 2B).

The biological accumulation factor (BCF), biological accumulation co-efficient (BAC), and Cd translocation factor (TF) also showed dose-dependent increases in response to Cd, and these values were further increased by VA supplementation (Figure 2C–E).

Phytochelatin (PC) content also showed a dose-dependent increase in response to Cd stress. Compared to the control, the PC content increased by 23% and 47% in response to exposure to 1.0 mM and 2.0 mM CdCl_2_, respectively. Vanillic acid supplementation further increased the PC content at both Cd doses (Figure 2F).

### 2.3. Vanillic Acid Prevented theLoss ofLeaf Relative Turgidity, Proline Accumulation, and Loss of Photosynthetic Pigment under CdToxicity

**Figure 3:**
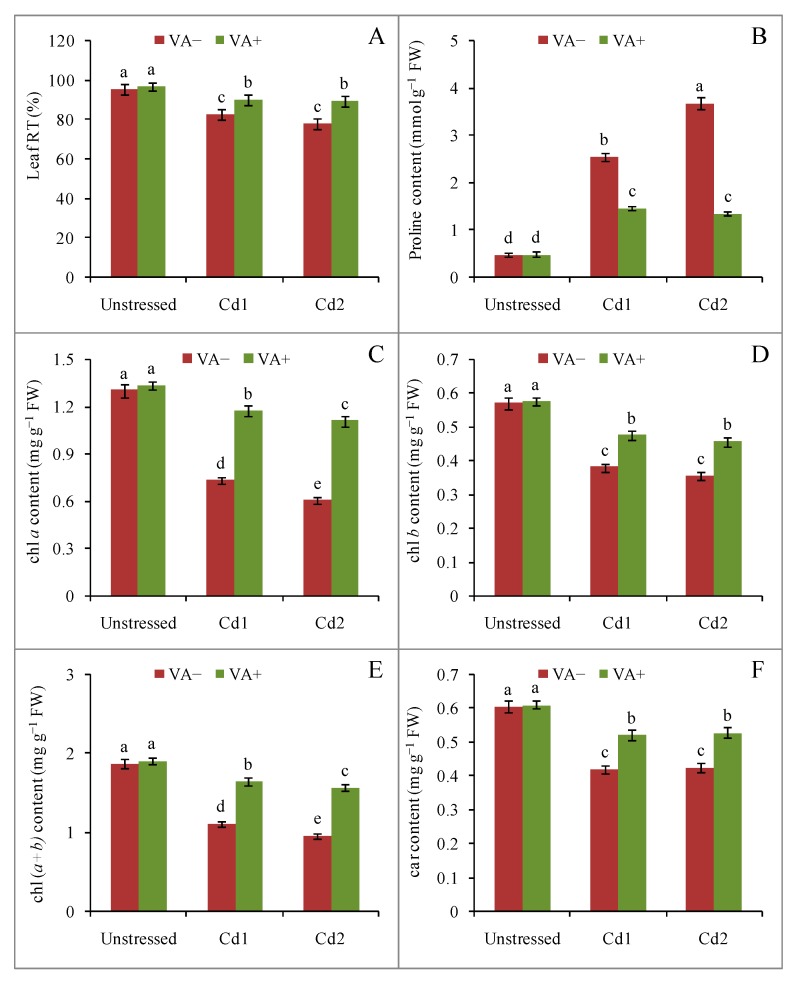
Effects of exogenous VA on leaf turgidity (RT) (%) (**A**), pro content (**B**), chlorophyll (chl) *a* (**C**), chl *b* (**D**), chl (*a* + *b*) (**E**), and carotenoid (car) (**F**) contents of rice (*Oryza sativa* L. cv. BRRI dhan54) seedlings under Cd toxicity stress. Mean (± SD) was computed from three replications of each treatment. Bars with dissimilar letters are significantly different at *p* ≤ 0.05 from Fisher’s least significant difference (LSD) test.

Cadmium stress altered the leaf turgidity (RT) of the rice seedlings. Compared to control leaves, leaf RT was reduced by 13% and 18% at mild and severe Cd stress, respectively. Treatment with VA in the presence of Cd suppressed the losses in RT, and thus RT values were higher by 9% and 14%, respectively, compared to the respective stress treatments (Figure 3A). Cadmium toxicity promoted osmolyte accumulation in the rice seedlings leaves, as indicated by a huge increase in proline (pro) content, but treatment with VA suppressed the Cd-induced accumulation of pro (Figure 3B).

Cadmium toxicity stress also caused a loss of photosynthetic pigments and induced chlorotic symptoms (Figure 1). Compared to the control seedlings, both mild and severe Cd stress caused a sharp decrease in chlorophyll (chl) *a*, chl *b*, and carotenoids (car), whereas VA treatment in the presence of Cd suppressed these losses of pigments (Figure 3C–F).

### 2.4. Vanillic Acid Suppressed the Induction of Oxidative Stress Markers under Cd Toxicity

**Figure 4:**
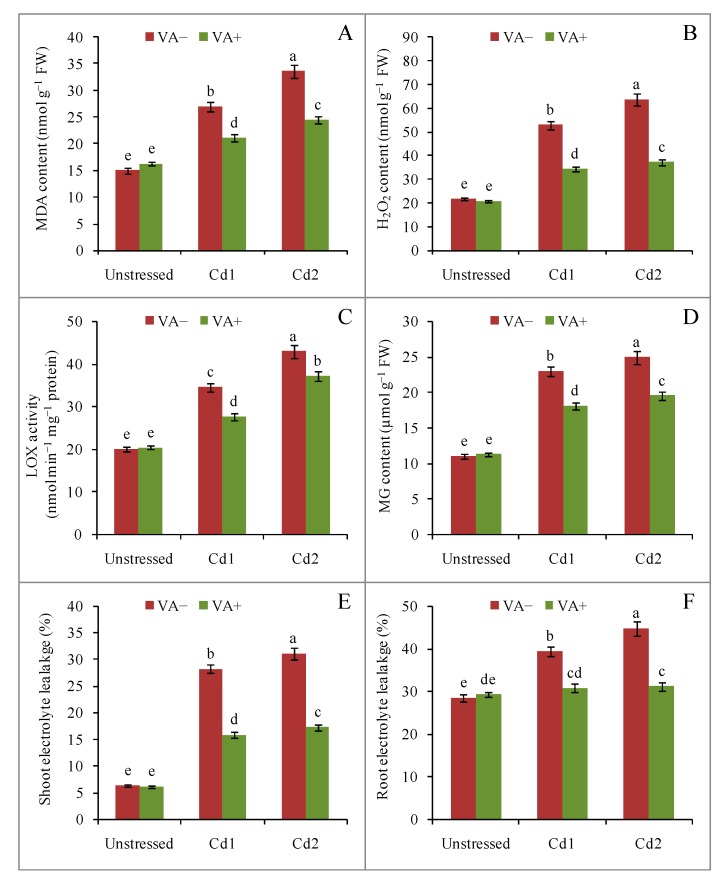
Effects of exogenous VA on malondialdehyde (MDA)(**A**) and H_2_O_2_ (**B**) content, lipoxygenase (LOX) activity (**C**), methylglyoxal (MG) content (**D**), and electrolyte leakage (EL) of shoot (**E**) and root (**F**) of rice (*Oryza sativa* L. cv. BRRI dhan54) seedlings under Cd stress. Mean (±SD) was computed from three replications of each treatment. Bars with dissimilar letters are significantly different at *p* ≤ 0.05 from Fisher’s least significant difference (LSD) test.

Malondialdehyde (MDA), which is produced as a byproduct of lipid peroxidation, was increased in the leaf tissue at both doses of Cd. Compared to the control seedlings, MDA content increased by 80% and 124% in response to mild (1.0 mM CdCl_2_) and severe (2.0 mM CdCl_2_) Cd stress, respectively. However, VA supplementation in the presence of Cd suppressed this increase in MDA content compared with the respective stressed seedlings (Figure 4A).

Similar to MDA, H_2_O_2_ content increased noticeably in rice leaf tissue upon Cd exposure (Figure 4B). Compared with the control seedlings, H_2_O_2_ content increased by 143% and 193% in mild (1.0 mM CdCl_2_) and severe (2.0 mM CdCl_2_) Cd stress-exposed seedlings, respectively. However, VA supplementation in the presence of Cd suppressed this H_2_O_2_production by 21% and 27%, respectively, compared with the respective Cd-stressed seedlings (Figure 4B).

In line with MDA and H_2_O_2_ content and lipoxygenase LOX activity increased noticeably in rice leaf tissue upon Cd exposure (Figure 4C). In comparison with the control seedlings, LOX activity increased by 72% and 114% following mild (1.0 mM CdCl_2_) and severe (2.0 mM CdCl_2_) Cd stress, respectively. Vanillic acid supplementation in the presence of Cd suppressed this increase in LOX activity by 19% and 13%, respectively, compared with their respective stressed seedlings (Figure 4C). In a similar way, the toxic MG content was increased due to Cd toxicity in a dose-dependent way, which was further suppressed by VA supplementation in the presence of Cd under both doses of Cd toxicity stress (Figure 4D).

Lipid peroxidation caused electrolyte leakage (EL) from the leaf and root tissue. In comparison with control shoots, EL increased by 346% and 391% in the 1.0 mM CdCl_2_ and 2.0 mM CdCl_2_ treatments, respectively. Conversely, VA supplementation in the presence of Cd suppressed the EL by 43% and 44%, respectively, compared with their respective stressed seedlings (Figure 4E).

Similarly, the root EL increased by 38% and 57% in 1.0 mM CdCl_2_ and 2.0 mM CdCl_2_-exposed seedlings, respectively, whereas VA supplementation in the presence of Cd suppressed EL by 38% and 30%, respectively, compared with their respective cadmium-stressed seedlings (Figure 4F).

### 2.5. Vanillic Acid Suppressed Changes in Nonenzymatic Antioxidant Content due toCd Toxicity

**Figure 5:**
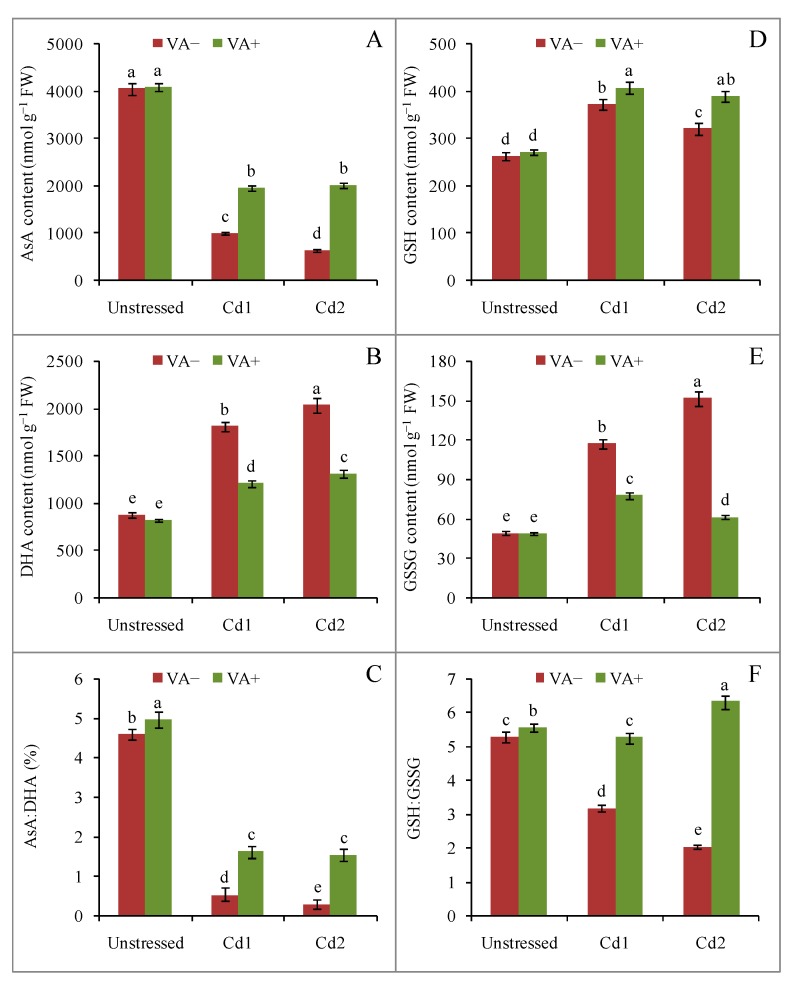
Effects of exogenous VA on ascorbic acid (AsA) (**A**) and dehydroascorbate (DHA)(**B**) contents, AsA/DHA ratio (**C**), reduced glutathione (GSH) (**D**) and oxidized glutathione (GSSG) (**E**) contents, and GSH/GSSG ratio (**F**) of rice (*Oryza sativa* L. cv. BRRI dhan54) seedlings under Cd stress. Mean (±SD) was computed from three replications of each treatment. Bars with dissimilar letters are significantly different at *p* ≤ 0.05 from Fisher’s least significant difference (LSD) test.

Among the nonenzymatic antioxidants, AsA content decreased by 75% and 84% due to mild and severe Cd toxicity, respectively, in comparison with control seedlings (Figure 5A). By contrast, the dehydroascorbate (DHA)content increased by 106% and 131% under mild and severe Cd stress, respectively (Figure 5B). Therefore, the redox pair ratio of AsA/DHA was reduced by 87% and 93% due to mild and severe Cd stress, respectively (Figure 5C). Vanillic acid supplementation in the presence of Cd suppressed the AsA decrease and the DHA content increase, leading to higher AsA/DHA ratios under both mild and severe Cd toxicity compared to their respective stress treatments (Figure 5A–C).

Although a sharp decrease in AsA content was observed due to Cd toxicity, both GSH (41% and 21%) and oxidized glutathione (GSSG) (136% and 206%) content increased under mild and severe Cd toxicity, respectively, in comparison with the control seedlings (Figure 5D,E). However, the redox couple ratio GSH/GSSG was reduced to 39% and 61%, respectively, under mild and severe Cd toxicity when compared to the control (Figure 5F). Addition of VA in the presence of Cd further increased the GSH content, but strongly suppressed the GSSG increase induced by Cd stress, resulting in an increase in the redox ratio for GSH/GSSG compared with their respective stress treatments (Figure 5D–F).

### 2.6. Vanillic Acid Induced Antioxidant Enzyme Activity under Cd Toxicity

**Figure 6:**
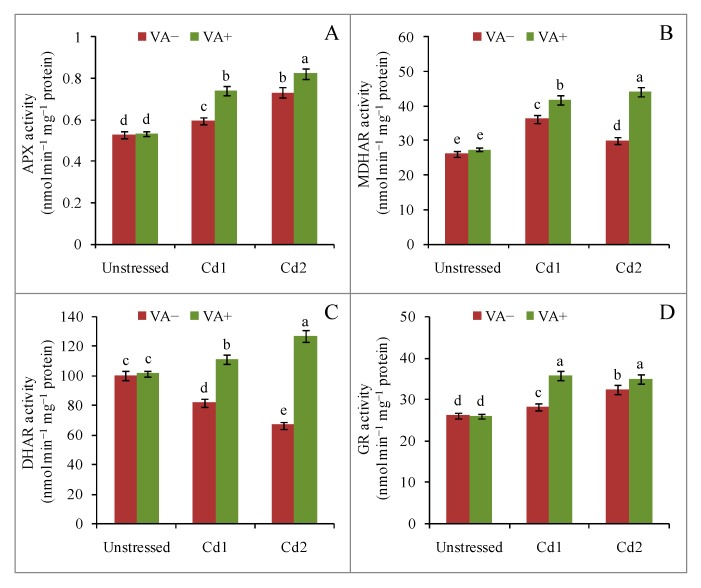
Effects of exogenous VA on activities of ascorbate peroxidase (APX) (**A**), monodehydroascorbate reductase (MDHAR) (**B**), dehydroascorbate reductase (DHAR) (**C**), and glutathione reductase (GR) (**D**) activity of rice (*Oryza sativa* L. cv. BRRI dhan54) seedlings under Cd toxicity stress. Mean (±SD) was computed from three replications of each treatment. Bars with dissimilar letters are significantly different at *p* ≤ 0.05 from Fisher’s least significant difference (LSD) test.

Among the antioxidant enzymes, the activity of the potent H_2_O_2_scavenger APX was increased by 12% and 39% by mild and severe Cd toxicity, respectively, compared to the control. Vanillic acid application in the presence of Cd promoted a further increase in the APX activity, by 24% and 12%, compared to the respective stress treatments (Figure 6A). Similar to the effect on APX activity, MDHAR activity was increased due to Cd toxicity, but the effect was greater for mild Cd stress (38%) than for severe Cd stress (15%). Vanillic acid supplementation in the presence of Cd further increased MDHAR activity by 15% and 46% under mild and severe Cd stress, respectively, compared to their respective control seedlings (Figure 6B). In contrast to the effects on APX and MDHAR activity, DHAR activity was decreased under both mild (18%) and severe (33%) Cd stress, respectively, compared to control, whereas VA supplementation in the presence of Cd increased the DHAR activity by 35% and 90% under mild and severe Cd stress, respectively, compared to their respective Cd stress treatments (Figure 6C). The GR activity was also increased (7% and 23%) under mild and severe Cd toxicity, respectively. However, a substantial increase (26% and 8%) in GR activity was observed under mild and severe Cd toxicity, respectively, by VA in the presence of Cd compared to their respective stressed seedlings (Figure 6D).

**Figure 7:**
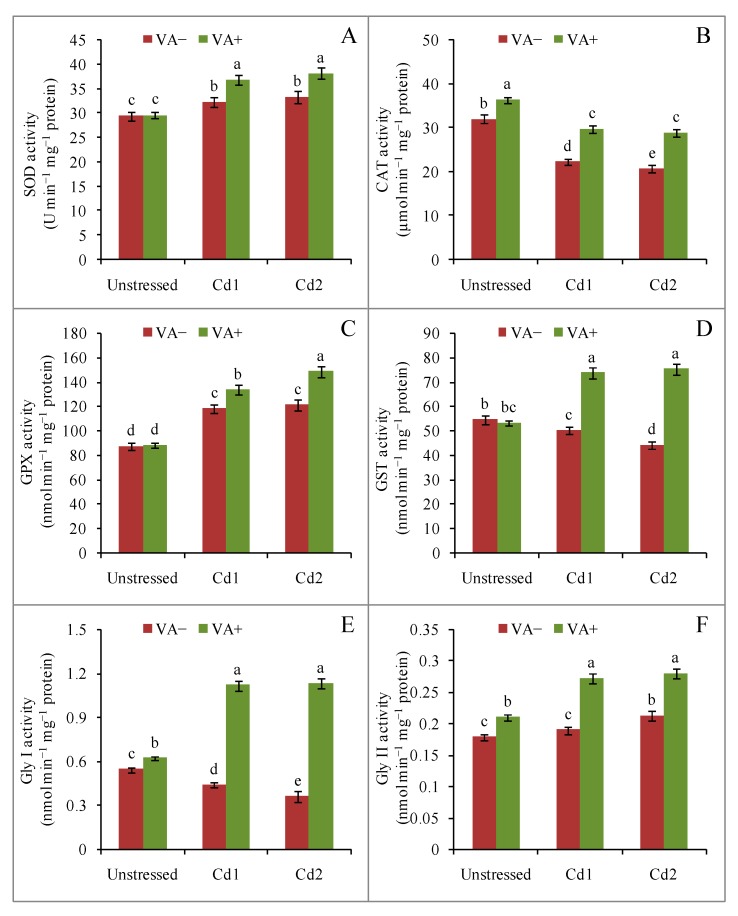
Effects of exogenous VA on activities of superoxide dismutase (SOD) (**A**), catalase (CAT) (**B**), glutathione peroxidase (GPX) (**C**),glutathione-*S*-transferase (GST) (**D**),glyoxalase I (Gly I) (**E**) and glyoxalase II (Gly II) (**F**) activity of rice (*Oryza sativa* L. cv. BRRI dhan54) seedlings under Cd toxicity stress. Mean (± SD) was computed from three replications of each treatment. Bars with dissimilar letters are significantly different at *p* ≤ 0.05 from Fisher’s least significant difference (LSD) test.

Mild and severe Cd toxicity increased the activities of the antioxidant enzymes SOD (9% and 13%, respectively) and GPX (35% and 38%, respectively) compared to control seedlings. Vanillic acid supplementation in the presence of Cd further increased SOD (9% and 14%) and GPX (12% and 22%) activity compared to their respective stress treatments (Figure 7A,C). By contrast, CAT activity decreased by 30% and 35%, due to mild and severe Cd stress, respectively, but VA supplementation in the presence of Cd suppressed the losses in CAT activity under both mild (33%) and severe (39%) Cd toxicity, respectively, compared to the respective stress treatments (Figure 7B). The activity of GST showed no changes under control and VA supplementation conditions, but GST activity was reduced by Cd stress, where 46% and 70% increases of GST activity was observed under both mild and severe Cd stresses, respectively, in the presence of VA (Figure 7D).

### 2.7. Vanillic Acid induced Glyoxalase Enzyme Activity under Cd Toxicity

Glyoxalase I activity was reduced by 19% and 34% under mild and severe Cd toxicity (Figure 7E), respectively, whereas Gly II activity increased by 6% and 18% (Figure 7F). Therefore, MG content increased under both mild and severe Cd toxicity, respectively (Figure 4D). Vanillic acid supplementation in the presence of Cd greatly increased Gly I activity by 154% and 213% following mild and severe Cd stress, respectively, and Gly II activity was also increased by 43% and 31% under both mild and severe Cd toxicity, respectively, compared to the respective stress treatments (Figure 7E,F).

### 2.8. Vanillic Acid Induced Mineral Homeostasis under Cd Toxicity

**Figure 8:**
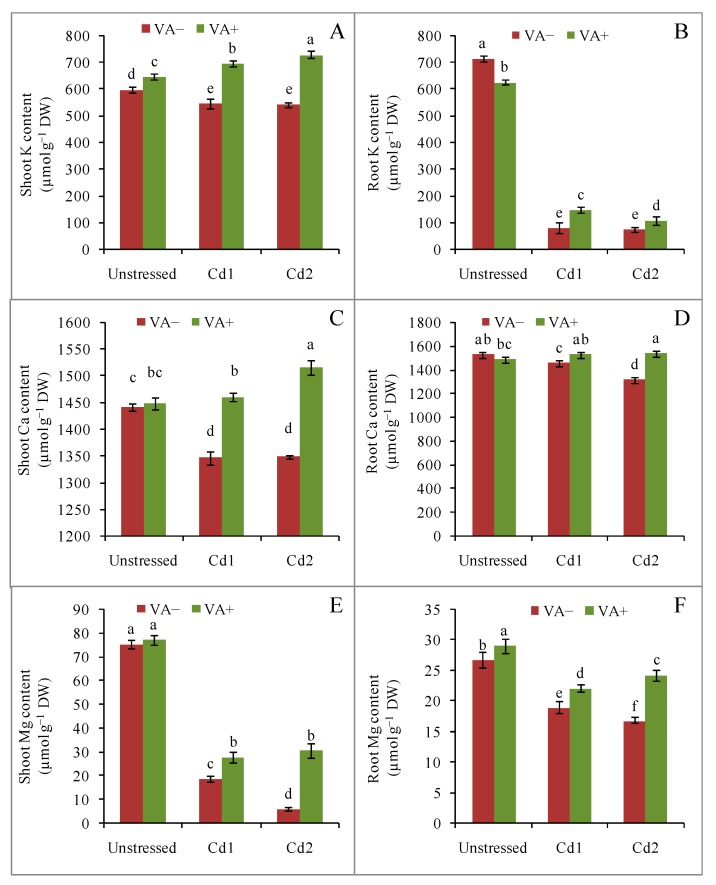
Effects of exogenous VA on the contents of shoot and root K, Ca, and Mg((**A**) shoot K; (**B**) root K; (**C**) shoot Ca; (**D**) root Ca; (**E**) shoot Mg; (**F**) root Mg) of rice seedlings under Cd toxicity stress. Mean (± SD) was computed from three replications of each treatment. Bars with dissimilar letters are significantly different at *p* ≤ 0.05 from Fisher’s least significant difference (LSD) test.

Shoot and root K content was decreased upon Cd toxicity stress, but the losses were suppressed by VA supplementation in the presence of Cd (Figure 8A,B). Similarly, shoot and root Ca content decreased under Cd stress, but VA supplementation in the presence of Cd suppressed both shoot and root Ca losses under both mild and severe Cd toxicity, with strong suppression observed in the shoot (Figure 8C,D). Shoot and root Mg content was reduced by Cd, but this effect was suppressed by VA supplementation, particularly in response to severe Cd stress (Figure 8E,F).

### 2.9. CorrelationAnalysis of the Different Vanillic Acid Responses

The correlation analyses showed that plant growth and physiological parameters were negatively correlated with oxidative stress markers (e.g., MDA and H_2_O_2_), whereas oxidative stress markers were negatively correlated with the components of the antioxidant defense system (Table S1a–c). Similarly, the glyoxalase system activity was negatively correlated with the MG content (Table S1c).

## 3. Discussion

Metal/metalloid contamination in the soil and environment has a negative effect on plants [16], as these compounds at high soil concentrations inhibit growth and disturb many biochemical processes, thereby leading to several negative consequences, particularly during the early stages of plant growth and development [17,18]. Cadmium is ubiquitous in many environments and highly toxic to all forms of life, and thus it causes significant yield losses due to its phytotoxicity [19]. By contrast, phenolic compounds, and especially low molecular weight organic acid (LMWOA) forms, are reported to alleviate the toxic effects of Cd stress [20]. As an LMWOA, VA has been shown to ameliorate the abiotic stress caused by drought and flooding [11,14]. Vanillic acid is also reported to increase plant growth and productivity, as well as upgrade the soil microbial community, in several test plant species [21,22,23]. However, a role for VA in ameliorating metal/metalloid toxicity, including Cd, has not been established. Therefore, the aim of the present study was to document the effects of exogenous VA on Cd toxicity responses in rice (*O. sativa* L. cv. BRRI dhan54) at the early seedling stage.

Cadmium causes several deleterious morphological effects at different stages of plant growth and development, including growth and biomass accumulation [3]. We observed a reduction in biomass under Cd stress in the present study. Inhibition of the supply of essential ions required for seedlingbiomass accumulation could be one of the reasons for the reduction in growth and biomass. Similar studies on growth parameters have also reported reduced growth and biomass accumulation in various plant species in response to Cd stress [24,25]. In the present study, the inclusion ofVA in the growth medium under Cd stress conditions prevented the suppression of biomass accumulation by Cd in rice seedlings. Previous reports have suggested that LMWOAs are capable of increasing growth and biomass under abiotic stresses, including Cd toxicity [24,25], indicating that they can alleviate the abiotic stress responses induced by metal/metalloids and allow greater tolerance to pollutants such as Cd.

Any agricultural strategy for increasing plant tolerance to Cd first requires an understanding of Cd accumulation as well as the Cd transport behavior inside plant organs. The recognition that LMWOAs are potent alleviators of metal/metalloid stress and that they can accumulate inside plant cells suggests that they have a dual role in regulating metal/metalloid stress in plants [24,25,26]. Similar to other previous findings, we also observed an increased accumulation of Cd in both shoots and roots of our rice seedlings, which showed a three- to fourfold greater increase in Cd content in the root compared with the shoot [24,25,26]. Interestingly, the VA treatment in the presence of Cd further increased the shoot and root Cd content. Previous studies have shown that application of LMWOAs in many plant species increased shoot and root metal/metalloid content, which might indicate metal scavenging as well as metal chelation and sequestration. Therefore, our findings are supported by previous findings of other researchers [24].

The visible symptoms of Cd stress in rice seedlings, including loss of biomass, were suppressed by the addition of VA despite the increased Cd accumulation in both roots and shoots. This suggested that even with high Cd accumulation, inclusion of VA in the presence of Cd in the growth medium alleviated Cd toxicity. Our findings also validated the results of other researchers [24]. Organic acids are renowned worldwide for their association with metal/metalloid stress tolerance in plants [24,25,26]. We also observed increases in the BCF and BAC values in response to VA in the cadmium-stressed rice seedlings. The reason for this might have been an increase in Cd solubility due to a decrease in pH by VA in the growth medium. Moreover, a VA-assisted release of strong ligands in the growth medium might assist in increased Cd uptake by the rice roots and subsequent translocation to the shoots [27]. Conversely, LMWOAs can chelate Cd to reduce the toxicity of free Cd in the growth medium [28]. Furthermore, the chelated intermediate might be translocated more efficiently through xylem [29]. Therefore, in our study, the observed effects of VA could indicate chelation of the free Cd in the growth medium, translocation to the growing shoots, and sequestration of the toxic Cd in cell vacuoles. In support of this statement, we also observed an increase in PC content under both doses of Cd, which further increased sharply by inclusion of VA in the growth medium, in agreement with previous findings [28]. Previous reports have suggested that PC, an oligomer synthesized in the plant cell in response to metal toxicity, acts as a binder for metals and transfers them for sequestration away from cell metabolic activities [30]. The activity of PC synthase in plant cells, as well as the formation of PCs, have been positively correlated with free Cd levels that can cause phytotoxicity symptoms [31]. The observed increase in PC content due to VA could further aid in ensuring plant tolerance and survivability, while also reducing oxidative stress, under Cd toxicity.

Plants exposed to metal/metalloid stress show a range of secondary stress symptoms, especially osmotic changes [32]. Therefore, plants also respond in various ways to mitigate water balance to cope with abiotic stresses. For example, osmotic adjustment and regulation of water content or water potential are some of the adaptation mechanisms used by plants to tolerate Cd exposure [33]. In our study, in response to Cd exposure, rice seedlings also showed lower water content under both mild and severe stress, in agreement with previous reports [24,33]. However, application of exogenous VA in the presence of Cd suppressed the changes in water balance seen in Cd-stressed seedlings in our study, hence suggested the role of LMWOAs in increasing water content in leaf tissues of cadmium-stressed plants. Besides maintaining water status, the biosynthesis of osmolytes is one of the vital strategies used by plants to mitigate the water balance changes under stress conditions. For example, biosynthesis and accumulation of proline, glycine betaine (GB), and trehalose (Tre) allows osmotic adjustment of Na^+^ stress inside cells to maintain water balance [34]. We found an increased pro content in rice seedling leaf tissues under both mild and severe Cd stress, but this accumulation was suppressed by VA under both mild and severe Cd stress. The exogenous application of VA prevented the changes in water balance in cadmium-stressed rice seedlings so the plants could reduce the requirement for biosynthesis of pro.

Metal/metalloids hamper the growth and development of the plant but they also affect physiological processes. One of the most deleterious effects of metal/metalloid toxicity is the destruction of photosynthetic pigments. The biosynthesis of chl and its content decreases in many plant species in response to toxic metal/metalloid stress, including Cd stress [28,33]. Our results indicated a marked decrease in photosynthetic pigment content in terms of the chl (*a* + *b*) contentin rice seedling leaves under Cd stress, as well as a decrease in car content. These decreases in pigment content due to Cd stress might be linked with the inhibition of several enzymes, leading to a disruption of pigment biosynthesis. Some researchers have also hypothesized that peroxidative breakdown of photosynthetic pigments, as well as the lipids of the chloroplast membrane, occurs in response to abiotic stress due to increased generation of ROS [35]. However, this effect was suppressed when VA was included in the growth medium, in agreement with previous published findings [33]. Improved chl and car content might be associated with an increased sequestration of Cd or an increased biosynthesis and/or decrease in the destruction of pigment complexes [24,25,33].

Similar to other environmental stresses, Cd stress mediates an enhanced generation of ROS, including O_2_^•−^, OH, and H_2_O_2_ [3]. These ROS are potentially capable of triggering membrane lipid peroxidation, as well as damaging amino acids, proteins, nucleotides, and nucleic acids. Membrane damage resulting from lipid peroxidation produces MDA, a major indicator of oxidative stress [9]. In our experiment, H_2_O_2_ and MDA content, as well as LOX activity, were markedly increased by Cd stress in a dose-dependent fashion. Simultaneously, the EL also increased. These responses might be attributed to a significant increase in ROS due to Cd, and they may also be responsible for the observed increases in the activity of the enzymes responsible for the degradation of lipid peroxides, which represent the attempt to control membrane damage and EL. Thus, our results are in line with those of previous researchers who observed similar responses to Cd-induced toxicities [24]. Higher H_2_O_2_, MDA, and EL levels also indicate that the antioxidant system defenses may be inadequate. However, the inclusion of VA in the growth medium suppressed the production of MDA and H_2_O_2_, while increasing LOX activity and ameliorating the EL observed in the Cd-stressed seedlings. These responses most likely reflect an enhanced activity of the ROS scavenging antioxidant defense system in the VA-treated seedlings. These observations are also in line with previous work [24,28] that reported are version of Cd-induced damage by LMWOAs.

Plants have evolved various mechanisms for protection against abiotic stress. A prime example is the antioxidant system, which consists of various enzymes and other non-enzyme components that are distributed throughout cell components (chloroplast, cytoplasm, apoplast, mitochondria, peroxisomes, and membranes) and allow dissipation of overproduced ROS. The end result is the ability to acclimate to unfavorable environments and to maintain growth and physiological functions. The content of these non-enzymatic components (AsA and GSH) and the potent activities of antioxidant enzymes (SOD, CAT, APX, MDHAR, DHAR, GR, GPX, and GST) have been documented to modulate growth under different stress conditions, including Cd stress [24,33,36].

The AsA–GSH cycle, or the ‘Foyer–Halliwell–Asada’ pathway, operates to scavenge H_2_O_2_ in plant cells [37]. AsA functions as the major antioxidant for reducing H_2_O_2_, OH, O_2_^•−^, and ^1^O_2_ levels. We found significant decreases in AsA content in the cadmium-stressed plants when compared with the control plants, which was mainly due to an increase in APX activity and decreased DHAR activity. The MDHAR activity increased under mild Cd stress, but it was not sufficient to repress the AsA content. A similar decrease in AsA content was also reported in other crops [24,25,27]. The upregulation of APX activity was also previously reported [25]. However, inclusion of VA in the presence of Cd increased the AsA content, as well as APX and DHAR activity, in cadmium-stressed rice seedling leaf tissue. This finding suggests that exogenous VA might play vital roles in AsA regeneration, in agreement with the findings of other researchers working on various other phytoprotectant chemicals [25,27,28,33].

Glutathione is another major component in the AsA–GSH cycle and is critical for AsA regeneration, xenobiotic detoxification, and metal/metalloid sequestration, as well as other mechanisms involved in stress tolerance [15]. Glutathione is linked with the AsA–GSH cycle for detoxification of H_2_O_2_, and it can also detoxify H_2_O_2_ via GPX/GST and xenobiotics through GST. Glutathione is also vital for MG detoxification via the Gly I and Gly II enzymes and has signaling properties [9]. In the present study, we found increased GSH content under both mild and severe Cd stress, together with increased GR and GPX activity. These changes might reflect aCd stress response involving the conversion of GSH to GSSGfor AsA recycling, whereas GPX activity is simultaneously increased to scavenge the overproduced H_2_O_2_, in agreement withother published findings [28]. The addition ofVA to the growth medium in the presence of Cd allowed further increases in the GSH content under both mild and severe Cd stress while also increasing the GR activity to enhance the recycling of GSSG to GSH. By contrast, the GST activity decreased in VA-treated cadmium-stressed seedlings, perhaps because the accumulation of H_2_O_2_was prevented by the enhanced antioxidant activity in the cells.

Superoxide dismutase activity was also increased by Cd stress, but the increase was suppressed byVA application in the presence of Cd. Reports have suggested that SOD is the plant’s first-line defense for scavenging toxic O_2_^•−^ radicals and converting them to H_2_O_2_. Therefore, the increased SOD activity in our study might be attributed toan increased O_2_^•−^ content, which was suppressed by VA. Our findings are in line with those of other researchers [24], as many studies on plant responses to various stresses have shown significant alterations in CAT activity and have identified CAT as the most efficient H_2_O_2_ scavenging enzyme [38]. In the present study, the CAT activity decreased under Cd stress, in agreement with the findings of Praveen et al. [39]. However, adding VA in the presence of Cd suppressed the decrease in CAT activity, in accordance with the previous findings [25].

A VA-induced MG-mediated inhibition of glycation has been reported in animal cells [40], but no similar reports have been published for plant cells. In our study, Cd toxicity decreased the activity of Gly I but increased Gly II enzyme activity, thereby increasing the MG content in rice seedling leaf tissues. The inclusion of VA in the growth medium increased both the Gly I and Gly II enzyme activity, thus reducing the MG content and providing the seedlings with tolerance against MG-induced glycation as well as oxidative stress. The results of the present study are corroborated by those of other researchers who reported phytoprotectant-induced increases in the Gly enzyme activity and concomitant reduction in MG content [41,42].

One of the major consequences of metal/metalloid stress is nutrient deficiency, as metal/metalloids compete with essential mineral elements for uptake into the plant [43]. Nutrient homeostasis could therefore reduce metal/metalloid accumulation, and thereby their toxicity, to enhance many physiological mechanisms under Cd stress conditions. In our study, Cd toxicity stress altered the mineral homeostasis of rice seedlings. We observed Cd toxicity-related reductions in shoot and root K, Ca, and Mg content, which is corroborated by many previous studies [44,45] that have reported a hermetic effect of Cd on mineral homeostasis in different plant species. Cd-induced oxidative stress also damages the cell membrane, which might also lower the nutrient content in roots. Vanillic acid supplementation in the presence of Cd restored the nutrient balance, in line with the results of other researchers [24]. Therefore, VA-induced nutrient availability reduced the toxic effects of Cd accumulation and restored the plant’s ability to run its physiological mechanisms smoothly.

## 4. Materials and Methods

### 4.1. Plant Materials and Stress Treatments

Manually sorted surface sterilized (10 minutes with 1% sodium hypochlorite) rice (*Oryza sativa* L. cv. BRRI dhan54) seeds were soaked in deionized water (DH_2_O) for 48 h and kept moist for the following24 h for sprouting. Then, the sprouted seeds were sown in plastic pots (8 cm diameter, a volume of 250 mL) hydroponically and incubated(40h). Afterward, the pots with 25 germinated seeds were facilitated growing under controlled conditions (temperature 25 ± 2 °C; relative humidity 65%–70%; light 350 µmol photon m^−1^ s^−2^; 16/8 h day/night duration) for 12 days in a cultivation chamber. During the growing period, the seedlings were nourished with2500-fold diluted Hyponex nutrient solution (Hyponex, Japan) controlling pH 6.5–7.0. The nutrient solution was renewed after every 72 h. At 12 days, the seedlings were treated with Cd (1.0 and 2.0 mM) and VA (50 µM) for 72 h solely and in combination. Hence, the experiment consisted of six treatments fitted in a completely randomized design (CRD) with three repetitions and repeated thrice, maintaining the same conditions. Data were collected after the treatment period with standard methods described later.

### 4.2. Observation of Plant Growth and Biomass Accumulation

Growth and biomass accumulation under different treatments were observed by measuring the shoot and root length from bases of the seedling to the shoot and root tip, respectively, from 10randomly selected seedlings. After excision at the root and shoot junctions, the fresh weight (FW) of shoot and root were weighed and mean FW value was expressed as mg seedling^−1^. Afterwards, the shoots and roots were dried separately for 48 h at 60°C and weighed again; mean dry weight (DW) value was expressed as mg seedling^−1^.

We measured the relative elongation of shoot and root elongation of different treatment combinations according to the procedure stated by Song et al. [46]. Briefly, before exposure to different treatments, a set of 10 randomly selected seedlings was measured for shoot and root length, expressed as Di. After harvesting, the same seedlings were again measured for final shoot and root length, expressed as Df. The initial shoot or root and final shoot or root length of the control seedlings were measured as DCi and DCf, respectively. The following equation was employed for calculating the relative elongation of shoot or root and expressed as a percentage:

$$\mathrm{Relative}\;\mathrm{elongation}\;=\frac{Df-Di}{DCi-DCf}\times 100$$

### 4.3. Determination of Cd and Other Nutrient Contents, and Measuring BAC, BCF, and TF of Cd

After acid digestion (HNO_3_:HClO_4_ at 5:1, *v*/*v*) of dried root and shoot, an atomic absorption spectrophotometer (AAS) was employed for measuring the content of Cd, K, Ca, and Mg present in the shoot and root according to Zasoski et al. [47].

Biological accumulation coefficient indicates the ratio of Cd content of the shoots to the growing media, and BCF indicates the ratio of Cd content of the roots to the growing media, whereas TF indicates the ratio of shoot Cd and root Cd content of rice seedlings. Therefore, BAC, BCF, and TF of Cd were calculated following the equations below [48]:

$$\mathrm{BAC}\;=\;\frac{Cd\;content\;in\;shoot}{Cd\;content\;in\;the\;growing\;media}$$

$$\mathrm{BCF}\;=\;\frac{Cd\;content\;in\;root}{Cd\;content\;in\;the\;growing\;media}$$

$$\mathrm{TF}\;=\;\frac{Cd\;content\;in\;shoot}{Cd\;content\;in\;root\;}$$

### 4.4. Determination of Stress Markers, Photosynthetic Pigment, Relative Turgidity, and Proline Content

We estimated the MDA content, following Heath and Packer [49], as the thiobarbituric acid (TBA) reactive substances. First, fresh harvested rice leaves (0.5 g) were extracted by 5% trichloroacetic acid (TCA) using a chilled mortar and pestle, and subsequent centrifugation was then conducted at 11,500× *g* for 15 min. The supernatants were mixed with TBA for reaction to obtained the MDA, which was further determined from the optical absorbance difference between 532 and 600 nm and calculated using an extinction coefficient of 155 mM^−1^ cm^−1^ expressed as nmol g^−1^ FW.

The method described by Alexieva et al. [50] with little modification was employed for the determination of H_2_O_2_ content. Briefly, fresh leaf samples were homogenized in 3 ml 5% TCA and centrifuged (11,500× *g* for 15 min). The supernatant was collected. An aliquot of 0.5 ml supernatant was mixed with 0.5 ml of potassium phosphate (K–P) buffer (0.5 M, pH 7.0) and 1 mL of 1M potassium iodide (KI), and the mixture was allowed for reaction to occur for 1 h in darkness. Absorbance was measured at 390 nm using standard curve and expressed as nmol g^−1^ FW.

For determining the amount of photosynthetic pigments,0.25g of fresh leaf samples were taken and emerged in 10 mL 80% ethanol and heated for 1h in a hot water bath at 60 °C. The absorbance of the colored solution was read in a spectrophotometer under 663, 645, and 470 nm. Chlorophyll *a*, *b,* (*a* + *b*), and car contents were then calculated according to the equations described by Arnon [51] and Wellburn [52], respectively.

Further, we estimated RT (%) as depicted from Barrs and Weatherly [53] using the following equation:

$$\mathrm{RT}\;\left(\%\right)\;=\;\frac{\mathrm{leaf}\;\mathrm{fresh}\;\mathrm{weight}-\mathrm{Leaf}\;\mathrm{dry}\;\mathrm{weight}}{\mathrm{Leaf}\;\mathrm{turgid}\;\mathrm{weight}-\mathrm{leaf}\;\mathrm{dry}\;\mathrm{weight}}\times 100$$

Subsequently pro content was estimated according to Bates et al. [54]. Fresh leaves (100 mg) were extracted in 3% sulfosalicylic acid. To 1 mL of supernatant, 1 mL of acid ninhydrin and 1 ml of glacial acetic acid was added, followed by incubation at 100 °C for 1hour. Then, the mixture was cooled to terminate reaction and toluene was added and vortaxed to separate the toluene chromophore containing pro. Proline was assessed spectrophotometrically at 520 nm using L-proline (Wako, Japan) as standard.

Methylglyoxal estimation by *N*-acetyl-L-cysteine assay was performed following Wild et al. [55]. Fresh leaves (0.25 g) were mashed with 5% perchloric acid on an ice cold mortar and centrifuged at 11,000× *g* to remove the residue. The supernatant was mixed with charcoal for decolorization and subsequently neutralized by sodium carbonate. The neutralized supernatant was further used for *N*-acetyl-L-cysteine assay of MG estimation at a wavelength of 288 nm.

### 4.5. Nonenzymatic Antioxidant Assay

For the determination of the nonenzymatic antioxidants AsA and GSH, we extracted the tissue according to Kampfenkel et al. [56].The contents of total AsA, reduced AsA, total GSH, and GSSG were assayed spectrophotometrically. For the determination of AsA pool, aliquots of 200 µL of supernatants were neutralized with K–P buffer (0.5 M, pH 7.0). Further, in the aliquots of total AsA and reduced AsA, 0.1 M dithiothretitol (DTT) and DH_2_O was added, respectively. Then, total and reduced AsA was determined optically at 265 nm employing a previously formed standard curve, and dehydroascorbate (DHA) was calculated by subtraction of reduced AsA from total AsA. In a similar manner GSH pool was determined after neutralizing aliquots of 200 µL supernatants with 0.5 M K–P buffer (pH 7.0) [57]. We determined GSSG after masking reduced GSH by 2-vinylpyridine. After that, total GSH and GSSG were assayed on the basis of the enzymatic recycling, where reduced GSH wasoxidized by 5,5-dithio-bis (2-nitrobenzoic acid, DTNB) in the presence of GR and reduced nicotinamide adenine dinucleotide phosphate (NADPH). The rate of absorption change was read at 412 nm optically, and previously prepared standard curves of GSH and GSSG were employed to determine the content. Finally, the reduced portion of GSH was calculated after subtracting GSSG from total GSH [58].

### 4.6. Protein, Antioxidant, and other Enzyme Activity Assay

Crude enzyme extract was prepared for assaying enzymatic activity where freshly harvested 0.5 g of wheat leaf samples were grinded in 1 ml ice cold extraction buffer that contained AsA (1 mM), K–P buffer (50 mM, pH 7.0), KCl (100 mM), β-mercaptoethanol (5 mM), and glycerol (10%, *w*/*v*). The homogenates were centrifuged at 11,500× *g* for 10 min, and the supernatants were collected and preserved (−60 °C). The experimental condition was maintained at 0–4°C temperature under controlled condition.

Protein quantity present in each sample was determination according to Bradford [59]. Proteins formed complexes with Coomassie Brilliant Blue dye, which was read optically at 595 nm. The amount of protein was then calculated using standard curve constructed with protein standard—bovine serum albumin (BSA).

Lipoxygenase (EC: 1.13.11.12) activity was assayed according to Doderer et al. [60] using linoleic acid as a substrate by observing the increase of absorbance at 234 nm and calculated using 25 mM^−1^ cm^−1^ as extinction coefficient.

Ascorbate peroxidase (EC: 1.11.1.11) activity was determined as stated by Nakano and Asada [61], where the assay mixture included K–P buffer (50 mM, pH 7.0), ethylenediaminetetraacetic acid (EDTA) (0.1 mM), AsA (0.5 mM), and H_2_O_2_ (0.1 mM). After adding H_2_O_2_, the reaction was started, and finally the activity of APX was computed using 2.8 mM^−1^ cm^−1^ as extinction coefficient.

Monodehydroascorbate reductase (EC: 1.6.5.4) activity was assayed following Hossain et al. [62] optically at 340 nm, using 703.4 µL of reaction mixture consistingof Tris-HCl buffer (50 mM, pH 7.5), AsA (2.5 mM), NADPH (0.2 mM), and ascorbate oxidase (AO) (0.5 unit), and was computed using 6.2 mM^−1^ cm^−1^ as extinction coefficient.

Dehydroascorbate reductase (EC: 1.8.5.1) activity wasrecorded with the method of Nakano and Asada [61], where the assay mixture contained K–P buffer (50 mM, pH 7.0), GSH (2.5 mM), EDTA (0.1 mM), and DHA (0.1 mM). Activity of DHAR was assayed from the increase in absorbance at 265 nm with the reduction of DHA for 1min. Finally, the activity of DHAR was calculated using 14 mM^−1^ cm^−1^ as extinction coefficient.

Glutathion reductase (GR; EC: 1.6.4.2) activity was measured according to Foyer and Halliwell [63] by observing the decline in absorbance at 340 nm, where reaction mixture consisted of K–P buffer (0.1 M, pH 7.0) and EDTA (1 mM). The reaction was NADPH-dependent and initiated with GSSG. Glutathione reductase activity was finally calculated using 6.2 mM^−1^ cm^−1^ as extinction coefficient.

Superoxide dismutase (SOD; EC 1.15.1.1) activity was determined on the basis of the reduction of nitro blue tetrazolium (NBT) using xanthine–xanthine oxidase system [64].

Catalase (CAT; EC: 1.11.1.6) activity was assayed following the method of Patra et al. [65] by observing the decrease in absorbance for 1 min (by conversion of H_2_O_2_ to water and O_2_) at 240 nm, where the enzyme extract was used to initiate the reaction. The activity of enzyme was computed using 39.4 M^−1^ cm^−1^ as extinction coefficient.

Glutathione *S*-transferase (GST; EC: 2.5.1.18) activity was spectrophotometrically measured according to Booth et al. [66], where the reaction mixture contained 1.5 mM GSH, 1 mM 1-chloro-2,4-dinitrobenzene (CDNB), and enzyme. The increase of absorbance was read at 340 nm for 1min and the enzyme activity was computed using the CDNB extinction coefficient of 9.6 mM^−1^ cm^−1^.

Glutathione peroxidase (GPX; EC: 1.11.1.9) activity was enumerated according to the procedure of Elia et al. [67], where the reaction buffer contained 100 mM K–P buffer (pH 7.0), 1 mM EDTA, 1mM sodium azide (NaN_3_), 0.12 mM NADPH, 2 mM GSH, and 1unit of GR. The reaction used 0.6 mM H_2_O_2_ as substrate, and the activity was calculated using extinction coefficient 6.6 mM^−1^ cm^−1^.

Glyoxalase I (Gly I; EC: 4.4.1.5) activity assay was recorded according to the method stated by Hossain et al. [68], where 700 µL of assay mixture consist of K–P buffer (100 mM, pH 7.0), magnesium sulfate (15 mM), GSH (1.7 mM), and MG (3.5 mM). After adding MG, the reaction began, and increase in absorbance was obtained at 240 nm for 1 min. The activity of Gly I was computed using 3.37 mM^−1^ cm^−1^ as extinction coefficient.

Glyoxalase II (Gly II; EC: 3.1.2.6) activity assay was performed as described by Principato et al. [69], where 500 µL of the reaction mixture contained Tris-HCl buffer (100 mM, pH 7.2), DTNB (0.2 mM), and S-D-lactoylglutathione (SLG, 1 mM). The reaction was initiated by adding SLG, and the increase in absorbance was recorded spectrophotometrically at 412 nm. Finally, the activity was computed using 13.6 mM^−1^ cm^−1^ as extinction coefficient.

### 4.7. Statistical Analysis

The data presented are the mean values of three replicates. The significant differences between treatments were statistically evaluated using Statistix10 software by one-way analysis of variance (ANOVA). Fisher’s least significant difference (LSD) test was used for comparison of means at the *p* < 0.05 level between treatments. Results, which were significantly different at *p* < 0.05, were marked by different letters in figures. Moreover, a correlation study was performed to infer the relationship between Cd toxicity and the VA supplementation under Cd toxicity stress. Furthermore, we showed the significant difference between sole Cd and VA supplementation with Cd using asterisks.

## 5. Conclusions

Cadmium toxicity stress strongly reduced growth and biomass accumulation in rice seedlings. The photosynthetic pigments were damaged by Cd exposure, and the osmotic status inside the cell was altered due to the toxic effects of Cd. Cadmium exposure promoted ROS generation and impaired the antioxidant defense and glyoxalase systems in rice seedlings. Vanillic acid supplementation in the presence of Cd in the growth medium noticeably suppressed the losses in the antioxidant defense and glyoxalase system and the ROS generation and lipid peroxidation observed in cadmium-stressed plants, while improving the osmotic status and preventing the loss of photosynthetic pigments. These results indicate that VA could serve as a phytoprotectant to reduce Cd toxicity stress in rice at the early seedling stage. Further study will be conducted to elucidate the molecular mechanism of VA-induced Cd stress tolerance, as well as to evaluate the practical application of VA in greenhouse and field conditions.

## Figures and Tables

**Table 1:** Effects of exogenous VA on plant height, root length, relative elongation of shoot and root, shoot and root fresh weight, and shoot and root dry weight of rice (*Oryza sativa* L. cv. BRRI dhan54) seedlings under Cd toxicity stress. C, VA, Cd1, and Cd2 indicate control, vanillic acid (50 µM), CdCl_2_ 1.0 mM, and CdCl_2_ 2.0 mM, respectively. RSE and RRE indicate relative elongation of shoot and relative elongation of root, respectively. Means (± SD) were calculated from three replications (*n* = 3) for each treatment. Values with different letters are significantly different at *p* ≤ 0.05 applying Fisher’s least significant difference (LSD) test.

Treatments	Plant Height (cm)	RSE (%)	Root Length (cm)	RRE (%)	Shoot FW (mg plant^−1^)	Root FW (mg plant^−1^)	Shoot DW (mg plant^−1^)	Root DW (mg plant^−1^)
**C**	15.5 ± 1.1 a	100.0 ± 0.0 b	7.1 ± 0.5 a	100.0 ± 0.0 b	66.4 ± 5.6 ab	36.7 ± 3.1 ab	10.8 ± 0.9 ab	6.6 ± 0.5 ab
**VA**	16.2 ± 1.3 a	113.5 ± 0.8 a	7.4 ± 0.4 a	106.8 ± 0.9 a	70.5 ± 5.3 a	38.2 ± 2.9 a	11.4 ± 0.8 a	6.8 ± 0.6 a
**Cd1**	13.4 ± 0.8 bc	46.1 ± 8.9 d	5.1 ± 0.3 bc	20.6 ± 6.2 e	54.5 ± 3.2 c	33.3 ± 1.9 b	8.8 ± 0.5 c	6.0 ± 0.4 b
**Cd1 + VA**	15.1 ± 0.2 ab	86.6 ± 6.7 c	5.6 ± 0.5 b	41.1 ± 0.4 d	61.3 ± 4.6 bc	36.1 ± 2.5 ab	9.9 ± 0.7 bc	6.5 ± 0.5 ab
**Cd2**	11.9 ± 1.1 c	12.1 ± 3.1 e	4.7 ± 0.4 c	6.1 ± 1.9 f	58.6 ± 4.5 bc	32.4 ± 2.7 b	9.5 ± 0.8 bc	5.8 ± 0.5 b
**Cd2 + VA**	15.1 ± 1.0 ab	88.0 ± 2.7 c	5.8 ± 0.5 b	45.9 ± 3.5 c	62.0 ± 3.5 bc	38.2 ± 2.1 a	10.1 ± 0.6 bc	6.8 ± 0.4 a

## Supplementary Materials

The following are available online at https://www.mdpi.com/2223-7747/9/2/188/s1, Table S1a: Correlation matrix of plant growth, osmotic status, photosynthetic pigments contents, Oxidative stress indicator, antioxidants and minerals contents, Table S1b. Correlation matrix of oxidative stress indicators, the AsA:GSH cycle and enzymatic antioxidants, Table S1c. Correlation matrix of the components of glyoxalase systems, Cd accumulation and translocation, other essential mineral components.
