# Supplementary material for: Modulation of Cadmium Tolerance in Rice: Insight into Vanillic Acid-Induced Upregulation of Antioxidant Defense and Glyoxalase Systems
Source: Plants (Basel). 2020 Feb 4;9(2):188. doi: 10.3390/plants9020188 (PMC7076431; doi:10.3390/plants9020188)
Supplement: Supplementary file 1 [file plants-09-00188-s001.pdf]

Table S1a. Correlation matrix of plant growth, osmotic status, photosynthetic pigments contents, Oxidative stress indicator, antioxidants and minerals contents

	Plant height	RSE	Root length	RRE	Shoot FW	Root FW	Shoot DW	Root DW	Leaf RWC	Pro	Chl *a*	Chl *b*	Chl (*a*+*b*)	car
Plant height	1													
RSE	0.887**	1												
Root length	0.852**	0.814**	1											
RRE	0.748**	0.871**	0.926**	1										
Shoot FW	0.554*	0.643**	0.684**	0.727**	1									
Root FW	0.588*	0.672**	0.505*	0.539*	0.865**	1								
Shoot DW	0.554*	0.643**	0.684**	0.727**	1.000**	0.865**	1							
Root DW	0.588*	0.672**	0.505*	0.539*	0.865**	1.000**	0.865**	1						
Leaf RWC	0.815**	0.919**	0.857**	0.892**	0.778**	0.745**	0.778**	0.745**	1					
Pro	−0.849**	−0.982**	−0.844**	−0.920**	−0.641**	−0.635**	−0.641**	−0.635**	−0.930**	1				
Chl *a*	0.845**	0.969**	0.833**	0.891**	0.719**	0.695**	0.719**	0.695**	0.963**	−0.971**	1			
Chl *b*	0.793**	0.911**	0.909**	0.967**	0.784**	0.646**	0.784**	0.646**	0.966**	−0.942**	0.960**	1		
Chl (*a*+*b*)	0.839**	0.963**	0.857**	0.916**	0.740**	0.689**	0.740**	0.689**	0.971**	−0.971**	0.998**	0.977**	1	
car	0.786**	0.902**	0.890**	0.943**	0.811**	0.689**	0.811**	0.689**	0.961**	−0.926**	0.962**	0.988**	0.975**	1
MDA	−0.801**	−0.933**	−0.851**	−0.934**	−0.608**	−0.521*	−0.608**	−0.521*	−0.895**	0.970**	−0.934**	−0.943**	−0.943**	−0.896**
H_2O_2	−0.837**	−0.972**	−0.863**	−0.942**	−0.671**	−0.607**	−0.671**	−0.607**	−0.929**	0.991**	−0.977**	−0.964**	−0.981**	−0.945**
LOX	−0.718**	−0.839**	−0.832**	−0.911**	−0.587*	−0.409 ns	−0.587*	−0.409 ns	−0.833**	0.884**	−0.853**	−0.906**	−0.871**	−0.833**
EL Shoot	−0.812**	−0.946**	−0.884**	−0.961**	−0.716**	−0.601**	−0.716**	−0.601**	−0.926**	0.972**	−0.972**	−0.980**	−0.980**	−0.970**
EL Root	−0.837**	−0.973**	−0.746**	−0.827**	−0.551*	−0.601**	−0.551*	−0.601**	−0.864**	0.973**	−0.956**	−0.871**	−0.943**	−0.865**
AsA	0.752**	0.874**	0.925**	0.994**	0.759**	0.573*	0.759**	0.573*	0.916**	−0.922**	0.910**	0.983**	0.934**	0.964**
DHA	−0.832**	−0.967**	−0.861**	−0.940**	−0.683**	−0.603**	−0.683**	−0.603**	−0.922**	0.983**	−0.979**	−0.964**	−0.983**	−0.951**
AsA:DHA	0.707**	0.824**	0.922**	0.990**	0.760**	0.523*	0.760**	0.523*	0.878**	−0.875**	0.862**	0.963**	0.892**	0.937**
GSH	−0.185 ns	−0.246 ns	−0.611**	−0.670**	−0.470 ns	−0.036 ns	−0.470*	−0.036 ns	−0.339 ns	0.347 ns	−0.297 ns	−0.530*	−0.354 ns	−0.500*
GSSG	−0.849**	−0.982**	−0.800**	−0.874**	−0.616**	−0.662**	−0.616**	−0.662**	−0.905**	0.988**	−0.962**	−0.903**	−0.955**	−0.905**
GSH:GSSG	0.802**	0.913**	0.629**	0.675**	0.551*	0.734**	0.551*	0.734**	0.833**	−0.880**	0.895**	0.761**	0.870**	0.801**
NPT	−0.145 ns	−0.195 ns	−0.567*	−0.623**	−0.373 ns	0.042 ns	−0.373 ns	0.042 ns	−0.294 ns	0.305 ns	−0.223 ns	−0.470*	−0.282 ns	−0.404 ns
PC	−0.095 ns	−0.131 ns	−0.484*	−0.532*	−0.250 ns	0.116 ns	−0.250 ns	0.116 ns	−0.229 ns	0.241 ns	−0.133 ns	−0.378 ns	−0.191 ns	−0.279 ns
SOD	−0.106 ns	−0.154 ns	−0.489*	−0.553*	−0.241 ns	0.154 ns	−0.241 ns	0.154 ns	−0.206 ns	0.258 ns	−0.150 ns	−0.385 ns	−0.206 ns	−0.291 ns
CAT	0.845**	0.951**	0.877**	0.921**	0.70^{8**}	0.608**	0.708**	0.608**	0.903**	−0.941**	0.954**	0.940**	0.958**	0.936**
GPX	−0.270 ns	−0.336 ns	−0.647**	−0.709**	−0.428 ns	−0.021 ns	−0.428 ns	−0.021 ns	−0.421 ns	0.433 ns	−0.366 ns	−0.587*	−0.420 ns	−0.501*
GST	0.448 ns	0.494*	0.061 ns	0.060 ns	0.129 ns	0.501*	0.129 ns	0.501*	0.382 ns	−0.409 ns	0.483*	0.231 ns	0.428 ns	0.297 ns

	Plant height	RSE	Root length	RRE	Shoot FW	Root FW	Shoot DW	Root DW	Leaf RWC	Pro	Chl *a*	Chl *b*	Chl (*a*+*b*)	car
APX	-0.272^{ns}	-0.332^{ns}	-0.576^{*}	-0.639^{**}	-0.286^{ns}	0.020^{ns}	-0.286^{ns}	0.020^{ns}	-0.383^{ns}	0.426^{ns}	-0.311^{ns}	-0.509^{*}	-0.360^{ns}	-0.390^{ns}
MDHAR	-0.004^{ns}	-0.034^{ns}	-0.451^{ns}	-0.494^{*}	-0.328^{ns}	0.145^{ns}	-0.328^{ns}	0.145^{ns}	-0.159^{ns}	0.140^{ns}	-0.097^{ns}	-0.355^{ns}	-0.158^{ns}	-0.300^{ns}
DHAR	0.685^{**}	0.771^{**}	0.409^{ns}	0.435^{ns}	0.392^{ns}	0.690^{**}	0.392^{ns}	0.690^{**}	0.676^{**}	-0.715^{**}	0.746^{**}	0.556^{*}	0.707^{**}	0.615^{**}
GR	-0.202^{ns}	-0.256^{ns}	-0.540^{*}	-0.603^{**}	-0.252^{ns}	0.045^{ns}	-0.252^{ns}	0.045^{ns}	-0.294^{ns}	0.357^{ns}	-0.213^{ns}	-0.429^{ns}	-0.265^{ns}	-0.332^{ns}
Gly I	0.450^{ns}	0.500^{*}	0.060^{ns}	0.068^{ns}	0.133^{ns}	0.474^{*}	0.133^{ns}	0.474^{*}	0.362^{ns}	-0.405^{ns}	0.486^{*}	0.231^{ns}	0.430^{ns}	0.300^{ns}
Gly II	0.158^{ns}	0.156^{ns}	-0.219^{ns}	-0.253^{ns}	0.010^{ns}	0.335^{ns}	0.010^{ns}	0.335^{ns}	0.061^{ns}	-0.031^{ns}	0.160^{ns}	-0.086^{ns}	0.103^{ns}	0.015^{ns}
MG	-0.763^{**}	-0.894^{**}	-0.902^{**}	-0.985^{**}	-0.698^{**}	-0.517^{*}	-0.698^{**}	-0.517^{*}	-0.888^{**}	0.939^{**}	-0.918^{**}	-0.971^{**}	-0.937^{**}	-0.942^{**}
BCF	-0.345^{ns}	-0.415^{ns}	-0.731^{**}	-0.786^{**}	-0.531^{*}	-0.154^{ns}	-0.531^{*}	-0.154^{ns}	-0.526^{*}	0.513^{*}	-0.448^{ns}	-0.670^{**}	-0.504^{*}	-0.604^{**}
BAC	-0.308^{ns}	-0.371^{ns}	-0.689^{**}	-0.742^{**}	-0.469^{*}	-0.110^{ns}	-0.469^{*}	-0.110^{ns}	-0.480^{*}	0.471^{*}	-0.389^{ns}	-0.616^{**}	-0.445^{ns}	-0.537^{*}
TF	-0.488^{*}	−0.579	-0.825^{**}	-0.889^{**}	-0.613^{**}	-0.278^{ns}	-0.613^{**}	-0.278^{ns}	-0.668^{**}	0.667^{**}	-0.610^{**}	-0.797^{**}	-0.658^{**}	-0.738^{**}
Cd Shoot	-0.368^{ns}	-0.431^{ns}	-0.632^{**}	-0.681^{**}	-0.389^{ns}	-0.099^{ns}	-0.389^{ns}	-0.099^{ns}	-0.524^{*}	0.509^{*}	-0.446^{ns}	-0.616^{**}	-0.489^{*}	-0.495^{*}
Root Cd	-0.439^{ns}	-0.509^{*}	-0.692^{**}	-0.743^{**}	-0.453^{ns}	-0.176^{ns}	-0.453^{ns}	-0.176^{ns}	-0.600^{**}	0.584^{*}	-0.526^{*}	-0.687^{**}	-0.567^{*}	-0.573^{*}
Shoot K	0.593^{**}	0.655^{**}	0.291^{ns}	0.297^{ns}	0.254^{ns}	0.504^{*}	0.254^{ns}	0.504^{*}	0.491^{*}	-0.566^{*}	0.628^{**}	0.407^{ns}	0.580^{*}	0.482^{*}
Root K	0.591^{**}	0.694^{**}	0.878^{**}	0.946^{**}	0.681^{**}	0.382^{ns}	0.681^{**}	0.382^{ns}	0.774^{**}	-0.777^{**}	0.747^{**}	0.893^{**}	0.786^{**}	0.854^{**}
Shoot Ca	0.686^{**}	0.781^{**}	0.476^{*}	0.508^{*}	0.414^{ns}	0.604^{**}	0.414^{ns}	0.604^{**}	0.652^{**}	-0.728^{**}	0.768^{**}	0.599^{**}	0.734^{**}	0.675^{**}
Root Ca	0.777^{**}	0.836^{**}	0.561^{*}	0.572^{*}	0.191^{ns}	0.407^{ns}	0.191^{ns}	0.407^{ns}	0.682^{**}	-0.826^{**}	0.772^{**}	0.626^{**}	0.743^{**}	0.612^{**}
Shoot Mg	0.737^{**}	0.841^{**}	0.929^{**}	0.993^{**}	0.721^{**}	0.518^{ns}	0.721^{**}	0.518^{*}	0.876^{**}	-0.896^{**}	0.860^{**}	0.955^{**}	0.888^{**}	0.922^{**}
Root Mg	0.774^{**}	0.930^{**}	0.854^{**}	0.946^{**}	0.620^{**}	0.528^{ns}	0.620^{**}	0.528^{*}	0.872^{**}	-0.948^{**}	0.915^{**}	0.921^{**}	0.923^{**}	0.918^{**}

**Significant at $P \le 0.01$; *Significant at $P \le 0.05$.

^{ns}Non−significant.

Table S1b. Correlation matrix of oxidative stress indicators, the AsA:GSH cycle and enzymatic antioxidants

	MDA	H_2O_2	LOX	Shoot EL	Root EL	AsA	DHA	AsA:DHA	GSH	GSSG	GSH:GSSG	SOD	CAT	GPX	GST	APX	MDHAR	DHAR	GR
MDA	1																		
H_2O_2	0.980**	1																	
LOX	0.968**	0.917**	1																
Shoot EL	0.965**	0.992**	0.909**	1															
Root EL	0.930**	0.964**	0.821**	0.935**	1														
AsA	−0.935**	−0.946**	−0.910**	−0.969**	−0.832**	1													
DHA	0.972**	0.998**	0.909**	0.995**	0.960**	−0.945**	1												
AsA:DHA	−0.906**	−0.909**	−0.907**	−0.940**	−0.768**	0.992**	−0.911*	1											
GSH	0.447^{ns}	0.411^{ns}	0.537**	0.493**	0.191^{ns}	−0.656**	0.421^{ns}	−0.727**	1										
GSSG	0.925**	0.969**	0.806**	0.947**	0.982**	−0.876**	0.963**	−0.818**	0.265^{ns}	1									
GSH:GSSG	−0.750**	−0.841**	−0.578*	−0.807**	−0.919**	0.689**	−0.837*	0.605**	0.039^{ns}	−0.934**	1								
SOD	0.421^{ns}	0.312^{ns}	0.570^{ns}	0.352^{ns}	0.098^{ns}	−0.519^{ns}	0.303^{ns}	−0.601**	0.891**	0.142^{ns}	0.212^{ns}	1							
CAT	−0.915**	−0.964**	−0.861**	−0.965**	−0.912**	0.920**	−0.971**	0.898**	−0.416^{ns}	−0.926**	0.819**	−0.282^{ns}	1						
GPX	0.588*	0.496*	0.726**	0.542*	0.275^{ns}	−0.691**	0.492*	−0.762**	0.914**	0.314^{ns}	0.030^{ns}	0.966**	−0.470*	1					
GST	−0.255^{ns}	−0.356^{ns}	−0.067^{ns}	−0.298^{ns}	−0.560*	0.091^{ns}	−0.358^{ns}	−0.020^{ns}	0.644**	−0.506*	0.757**	0.766**	0.337ns	0.626**	1				
APX	0.588*	0.460^{ns}	0.726**	0.468^{ns}	0.269^{ns}	−0.609**	0.439ns	−0.670**	0.763**	0.302^{ns}	0.039^{ns}	0.946**	−0.400^{ns}	0.946**	0.593**	1			
MDHAR	0.292^{ns}	0.217^{ns}	0.443^{ns}	0.299^{ns}	−0.021^{ns}	−0.481*	0.226ns	−0.573*	0.956**	0.031^{ns}	0.290^{ns}	0.933**	−0.220^{ns}	0.925**	0.804**	0.798**	1		
DHAR	−0.552*	−0.657**	−0.350^{ns}	−0.609**	−0.797**	0.455^{ns}	−0.653**	0.352^{ns}	0.310^{ns}	−0.798**	0.957**	0.478*	0.633**	0.314^{ns}	0.90^{3**}	0.299^{ns}	0.547*	1	
GR	0.483*	0.377^{ns}	0.593**	0.394^{ns}	0.183^{ns}	−0.562*	0.355ns	−0.627**	0.824**	0.254^{ns}	0.080^{ns}	0.961**	−0.329^{ns}	0.919**	0.674**	0.959**	0.828**	0.337^{ns}	1

**Significant at $P \leq 0.01$; *Significant at $P \leq 0.05$.

^{ns}Non-significant.

Table S1c. Correlation matrix of the components of glyoxalase systems, Cd accumulation and translocation, other essential mineral components

	Gly I	Gly II	MG	NPT	PC	BCF	BAC	TF	Shoot Cd	Root Cd	Shoot K	Root K	Shoot Ca	Root Ca	Shoot Mg	Root Mg
Gly I	1															
Gly II	0.899**	1														
MG	−0.135^{ns}	0.213^{ns}	1													
NPT	0.727**	0.871**	0.574*	1												
PC	0.782**	0.941**	0.486*	0.964**	1											
BCF	0.557*	0.764**	0.740**	0.966**	0.926**	1										
BAC	0.608**	0.815**	0.692**	0.968**	0.958**	0.993**	1									
TF	0.388^{ns}	0.647**	0.855**	0.905**	0.851**	0.981**	0.963**	1								
Shoot Cd	0.452^{ns}	0.719**	0.681**	0.796**	0.867**	0.865**	0.889**	0.860**	1							
Root Cd	0.387^{ns}	0.673**	0.743**	0.786**	0.844**	0.875**	0.892**	0.886**	0.995**	1						
Shoot K	0.936**	0.810**	−0.338^{ns}	0.521*	0.624**	0.342^{ns}	0.407^{ns}	0.164^{ns}	0.345^{ns}	0.269^{ns}	1					
Root K	−0.202^{ns}	−0.496*	−0.933**	−0.807**	−0.722**	−0.916**	−0.878**	−0.972**	−0.790**	−0.833**	0.012^{ns}	1				
Shoot Ca	0.834**	0.635**	−0.539*	0.293^{ns}	0.429^{ns}	0.106^{ns}	0.179^{ns}	−0.076^{ns}	0.201^{ns}	0.112^{ns}	0.942**	0.263^{ns}	1			
Root Ca	0.666**	0.300*	−0.624**	0.138^{ns}	0.135^{ns}	−0.056^{ns}	−0.039^{ns}	−0.227^{ns}	−0.184^{ns}	−0.249^{ns}	0.731**	0.362^{ns}	0.786**	1		
Shoot Mg	−0.006^{ns}	−0.330*	−0.976**	−0.675**	−0.596**	−0.828**	−0.791**	−0.919**	−0.730**	−0.788**	0.214^{ns}	0.966**	0.438^{ns}	0.535*	1	
Root Mg	0.288^{ns}	−0.026*	−0.936**	−0.420^{ns}	−0.317^{ns}	−0.599**	−0.549*	−0.732**	−0.489*	−0.560*	0.528*	0.812**	0.697**	0.706**	0.917**	1

**Significant at $P \leq 0.01$; *Significant at $P \leq 0.05$.

^{ns}Non−significant.
